# Supplementary material for: All-at-once RNA folding with 3D motif prediction framed by evolutionary information
Source: Nat Methods. 2025 Oct 3;22(10):2094–106. doi: 10.1038/s41592-025-02833-w (PMC12510878; doi:10.1038/s41592-025-02833-w)
Supplement: Supplementary file 3 — Source code to run CaCoFold-R3D. Data and results. [file 41592_2025_2833_MOESM3_ESM.gz › supplemental_material/results/CaCoFold-R3D/CaCoFold-R3D_Rfam/RF02209_WT1-AS_7.R2R.sto.pdf]

RF02209_WT1-AS_7

5′-RR●●R●●CYC●●R●CYY●●R○●RR●●●CYA●●RARGG●○YUY●RYRGY○CYRC●●CUAUUAUU●UACCGUAAAUCUUYUAAAUUCUGGAACUAAUUAYAUAGAGGAUAUGUCUCAAUUUGUUCUGCAUUAAUGCCACA●UGGG○AUG●RR●●●○G●CC●UR●CCRGARCAGAU●Y●YRRGCYCAUGAAAUUGAUGAACUGAGA●YUGCU●CYAGUCCUGARYRYACCAUCUGGAAUUCCAG●CUGAGGUUACAA●●AGARCUYCUGACCCCARAUUCAACUUU●C●AA●AAC
